# Small copepods could channel missing carbon through metazoan predation

**DOI:** 10.1002/ece3.4546

**Published:** 2018-10-30

**Authors:** Álvaro Roura, Jan M. Strugnell, Ángel Guerra, Ángel F. González, Anthony J. Richardson

**Affiliations:** ^1^ Departamento de Ecología y Recursos Marinos Instituto de Investigaciones Marinas (IIM, CSIC) Vigo Spain; ^2^ Centre for Sustainable Tropical Fisheries and Aquaculture James Cook University Townsville Queensland Australia; ^3^ Centre for Applications in Natural Resource Mathematics, School of Mathematics and Physics University of Queensland St Lucia Queensland Australia; ^4^ CSIRO Oceans and Atmosphere, Queensland Biosciences Precinct St Lucia Queensland Australia

**Keywords:** biogenic fluxes, biological pump, carbon sink, climate change, copepods, fisheries, global ecosystem models, trophic ecology, zooplankton

## Abstract

Global ecosystem models are essential tools for predicting climate change impacts on marine systems. Modeled biogenic carbon fluxes in the ocean often match measured data poorly and part of this could be because small copepods (<2 mm) are modeled as unicellular feeders grazing on phytoplankton and microzooplankton. The most abundant copepods from a seasonal upwelling region of the Eastern North Atlantic were sorted, and a molecular method was applied to copepod gut contents to evaluate the extent of metazoan predation under two oceanographic conditions, a trophic pathway not accounted for in global models. Scaling up the results obtained herein, based on published field and laboratory estimates, suggests that small copepods could ingest 1.79–27.20 gigatons C/year globally. This ignored metazoan‐copepod link could increase current estimates of biogeochemical fluxes (remineralization, respiration, and the biological pump) and export to higher trophic levels by 15.6%–24.4%. It could also account for global discrepancies between measured daily ingestion and copepod metabolic demand/growth. The inclusion of metazoan predation into global models could provide a more realistic role of the copepods in the ocean and if these preliminary data hold true at larger sample sizes and scales, the implications would be substantial at the global scale.

## 1 INTRODUCTION

A significant challenge in understanding how the oceans will respond to anthropogenic impacts is to quantify and model the role of ocean ecosystems in the global carbon cycle (Gattuso et al., 2015; Mitra, Castellani, et al., 2014; Pauly et al., 2002; Stock, Dunne, & John, 2014). Such global ecosystem models or end‐to‐end models have a modeling framework that encompasses complex physicochemical oceanographic processes operating simultaneously over different spatiotemporal scales and organisms ranging from microbes to whales (Rose et al., 2010; Travers, Shin, Jennings, & Cury, 2007). These models can be used as predictive tools for assessing impacts of climate change in the oceans (Blanchard et al., 2017; Gattuso et al., 2015), but there is a significant mismatch between measured and modeled carbon (Buitenhuis et al., 2006; Stock et al., 2014). The source of this discrepancy is unknown, but modeled carbon is underestimated in global ecosystem models. Current solutions to these discrepancies have been to increase the maximum copepod grazing rates by 30% compared with laboratory values to match the mesozooplankton observations (Hernández‐León & Ikeda, 2005) or consider an “artificial” (i.e. largely below the optimal prey/predator ratio) carbon ingestion coming from particulated organic carbon and bacteria (Buitenhuis et al., 2006). For example, in the COBALT model used to predict the fate of anthropogenic C, microzooplankton ingestion by mesozooplankton such as copepods was increased to >97% of its standing stock to artificially match observed and modeled mesozooplankton production (Stock et al., 2014). Global models thus omit a significant carbon source that might be channeled through copepod predation but has not yet been accounted for.

Zooplankton is mostly represented in marine ecosystem models by copepods, the most abundant oceanic metazoan (Rose et al., 2010; Travers et al., 2007). Copepods perform several crucial roles including grazing primary producers (PP) (Buitenhuis et al., 2006), as prey for higher trophic levels (Beaugrand, Brander, Alistair Lindley, Souissi, & Reid, 2003; Pauly et al., 2002), and sustaining phytoplankton production via ammonia excretion (Hernández‐León, Fraga, & Ikeda, 2008). Copepods are thus keystone components of the biological pump as they increase particulate organic carbon flux to deeper layers through sinking fecal pellets and carcasses and through diel vertical migration (Turner, 2015).

**Figure 1:**
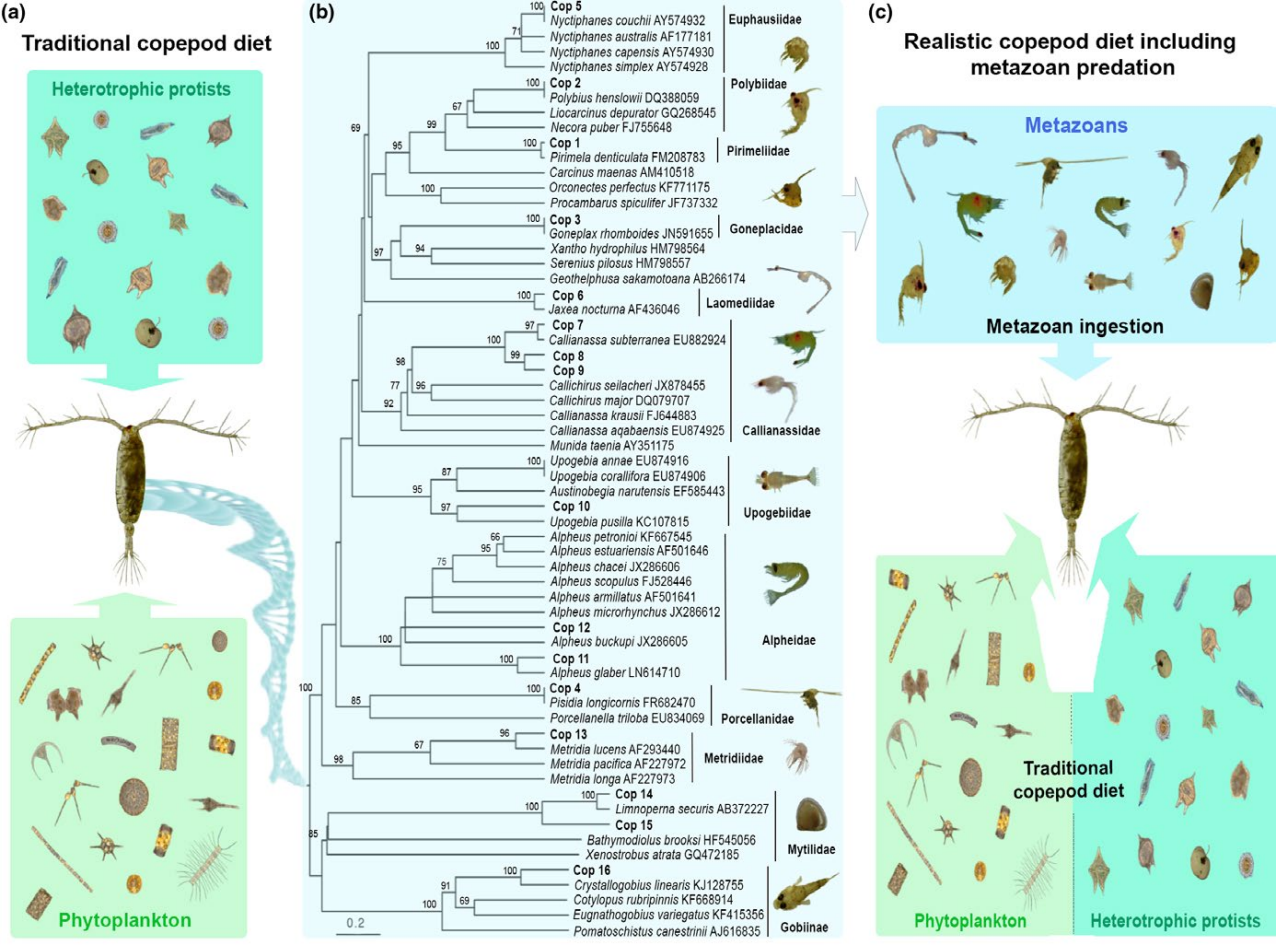
The changing paradigm in global ecosystem models. (a) Global ecosystem models consider small copepods as unicellular feeders preying upon phytoplankton (dark green) and heterotrophic protists (light green). (b) Metazoan prey detected in copepods with DNA‐based methods (Cop1–Cop 16, see Table 1 for species details) represented in a maximum‐likelihood tree associated with their closest matches. Bootstrap values above 60% after 1,000 replications are shown. (c) Realistic representation of copepod trophic links including the metazoan predation (in blue) could affect global estimates of carbon and nutrient fluxes in the pelagic realm. Photograph credits (organisms): Dr Isabel Teixeira (panel a), Alexandra Castro (copepod), and own material (panel b,c)

It is well known that several large copepods species prey upon smaller zooplankton including copepods (Kleppel, 1993; Turner, 2004), but it is less known that small copepods (<2 mm in size) apparently also ingest other small metazoans. The ingestion of metazoans by small copepods has been confirmed by visual analysis of gut contents in small calanoid (Paffenhöfer & Lewis, 1990), cyclopoid (Turner, 2004), and poecilostomatoid copepods (You‐Bong, Bong‐Cheol, & Makoto, 1998). Recently, molecular methods have also confirmed the detection of metazoan prey in small copepods in laboratory (Durbin, Casas, Rynearson, & Smith, 2008) and field studies (e.g. Cleary, Durbin, Rynearson, & Bailey, 2015; Hu et al., 2014; Yi et al., 2017). This information about the diet of small copepods could be important because they are typically modeled as unicellular feeders in global ecosystem models (Figure 1a), feeding upon phytoplankton (Paffenhöfer, 1988), ciliates, and heterotrophic dinoflagellates (Calbet & Saiz, 2005; Saiz & Calbet, 2007, 2011 ), but ignoring the potential carnivorous input from metazoans.

To make significant further progress in modeling mesozooplankton in global models, the missing carbon source associated with the food preference and grazing rate as a function of food quality in small copepods need to be quantified (Mitra, Castellani, et al., 2014; Rose et al., 2010). As this carnivorous predation in small copepods has been disregarded as an energy source in marine ecosystem models, here we: (a) test the extent of the metazoan pathway in the copepod community of a seasonal upwelling region under two oceanographic conditions; and (b) produce a preliminary estimate of how much carbon and nitrogen could be missing in the pelagic realm at a global scale if we ignore this trophic link.

## 2 MATERIAL AND METHODS

### 2.1 Zooplankton sampling

Zooplankton samples in this study are from long‐term sampling in the CAIBEX project off the Ría de Vigo, NW Iberian Peninsula (42°12.80′N, 09°00′W). They were collected at night with a Hydro‐Byos multinet of 200 µm mesh in July (13, 14, 20, and 21), September (22, 24, 27, and 30), and October 2010 (13 and 15). Four transects (T2–T5) were carried out each day, and two samples were collected on each transect: one close to the bottom (25, 30, 70 and 90 m depth on transects T2–T5, respectively) and the other close to the surface (1–4 m depth). Samples collected were fixed with 96% ethanol and stored at −20ºC.

Samples collected at the surface within the Ría de Vigo on July 14th and September 30th were selected for the following reasons: (a) samples belonged to the coastal mesozooplankton community, which is the community with highest diversity and abundance of small copepods (Roura et al., 2013); and (b) these samples reflected two contrasting oceanographic conditions (high and low productivity), which enabled testing of whether metazoan predation in copepods was influenced by the productivity of the system. We defined two productivity conditions: (a) downwelling (14/7/2010) defined by warm temperature (17.1°C), nutrient depleted surface waters, and low phytoplankton biomass (2.8 mg Chl‐*a*/m^3^), where more metazoan predation would be expected; and, (b) upwelling (30/09/2010), with cool temperature (13.9°C), nutrient‐enriched waters, and high phytoplankton biomass (9.2 mg Chl‐*a*/m^3^), where more grazing and less metazoan prey would be expected.

The dominant copepod species were sorted from samples after the downwelling event (seven species) and after the upwelling event (10 species, Table 1). We selected one adult female from each of the dominant species with sizes ranging from 0.8 to 2.7 mm length, commonly found in the zooplankton communities of upwelling ecosystems worldwide (Peterson, 1998): 15 calanoid species, of which 12 can be considered small (<2 mm total length: *Acartia clausii*,* Aetideus armatus*,* Centropages chierchiae*,* C. typicus*,* Clausocalanus* sp., *Ctenocalanus vanus*,* Diaixis pygmaea*,* Isias clavipes*,* Paracalanus parvus*,* Pleuromamma gracilis*,* Pseudocalanus elongatus*,* Temora longicornis*) and three large (>2 mm total length: *Calanoides carinatus*,* Calanus helgolandicus*,* Paraeuchaeta hebes*), and two small cyclopoids (*Oithona* sp. and the small poecilostomatoid *Corycaeus* sp). The cyclopoid and poecilostomatoid copepods were included as a positive control for the presence of metazoans in the diet since they are well‐known carnivores (Paffenhöfer & Lewis, 1990; Turner, 2004).

**Table 1:** Metazoan prey detected in copepods

Prey detected						Downwelling							Upwelling									
						s	s	s	s	s	s	l	s	s	s	s	s	s	s	s	l	l
OTUs Accession number	OTUs	Taxon	Top BLAST hit	Accession number	(%)	C1	C2	C3	C4	C5	C6	C7	C8	C9	C 10	Cl1	C12	C13	C14	C15	C16	C17
FR851241	Cop 1	Brachyura	*Pirimela denticulata*	FM208783	99		3	2	4			4	4		3	4	1	2	1	5	2	2
LN614707	Cop 2	Brachyura	*Polybius henslowii*	FM208765	100													1				
LN614708	Cop 3	Brachyura	*Goneplax rhomboides*	JN591655	100													2				
FR851242	Cop 4	Anomura	*Pisidia longicornis*	FR682470	100	3		2	1			1										2
FR851243	Cop 5	Euphausiacea	*Nyctiphanes couchii*	AY574933	97						1											
FR851244	Cop 6	Gebiidea	*Jaxea nocturna*	AF436046	98		1														2	
FR851245	Cop 7	Axiidea	*Callianassa subterranea*	EU882924	99												1					
FR851246	Cop 8	Axiidea	*Callianassa* sp.	EU882924	93												1					
FR851247	Cop 9	Axiidea	Callianassidae	EU882924	90												1					
FR851248	Cop 10	Gebiidea	Upogebiidae	EU874916	81																1	
FR851249	Cop 11	Caridea	*Alpheus glaber*	LN614710	96		1															
FR851250	Cop 12	Caridea	Alpheidae	FJ528445	78									4								
LN614709	Cop 13	Copepoda	*Metridia lucens*	AF293440	96	1																
FR851251	Cop 14	Bivalvia	*Limnoperna securis*	AB372227	98														2			
FR851252	Cop 15	Bivalvia	Mytilidae	AB372227	87														1			
FR851253	Cop 16	Teleostei	Gobiinae	EF218650	92					4												

Notes

Summary of the 16 (Cop1–Cop16, Figure 1b) prey or operational taxonomic units (OTUs) detected in the digestive tract of 17 different adult female copepod species collected during downwelling (C1–C7) and upwelling events (C8–C17) off the NW Iberian Peninsula. Numbers represent the prey detected on each copepod. Copepods are ordered by size under each oceanographic condition, with small copepods (<2 mm) labeled with an “s,” and large copepods (>2 mm) labeled with an “l.”

C1: *Diaixis pygmaea*; C2: *Clausocalanus* sp.; C3: *Temora longicornis*; C4: *Acartia clausii*; C5: *Isias clavipes*; C6: *Centropages chierchiae*; C7: *Paraeuchaeta hebes*; C8: *Oithona* sp.; C9: *Corycaeus* sp.; C10: *Paracalanus parvus*; C11: *Pseudocalanus elongatus*; C12: *Ctenocalanus vanus*; C13: *Aetideus armatus*; C14: *Centropages typicus*; C15: *Pleuromamma gracilis*; C16: *Calanoides carinatus*; C17: *Calanus helgolandicus*.

### 2.2 Molecular identification of metazoan prey in copepods

To avoid possible contaminants from the body surface, animals were washed with sterile distilled water, and appendages removed (mandibles, maxillules, maxillas, maxillipeds, and swimming legs). The digestive tract from each copepod species was dissected or suctioned with a pipette and the DNA extracted using the QIAamp DNA Micro Kit (QIAGEN), eluting the DNA in only 15 µl of ultrapure water. Metazoan DNA within the digestive tract of the copepods was amplified using a set of primers designed to target a broad spectrum of fishes, molluscs, echinoderms, and crustaceans (16Scruf 5′ GACGATAAGACCCTATAA 3′ and 16Scrur 5′ CGCTGTTATCCCTAAAGTAA 3′), which amplify a small region (around 200 bp) of the 16S rRNA gene located 200‐ to 240‐bp downstream of the universal primer 16Sar (5′ CGCCTGTTTATCAAAAACAT 3′, Simon et al., 1994). This set of primers was previously used to identify 20 different metazoan prey in *Octopus vulgaris*, but failed to amplify copepod DNA (Roura, González, Redd, & Guerra, 2012).

A semi‐nested PCR approach was employed to maximize the amount of prey DNA available. An initial PCR was carried out with the universal 16Sar and the primer 16Scrur to increase the proportion of prey DNA. The second PCR (semi‐nested) was performed with primers 16Scruf and 16Scrur to preferentially amplify the prey DNA. PCR reactions were performed in a final volume of 25 µl, containing 2.5 µl 10X PCR reaction buffer, 0.5 µl dNTPs (Roche), 0.75 µl each primer (10 µM), and 0.025 U/µl Taq polymerase (Roche). The first PCR contained 50–100 ng of template DNA and the nested PCR contained 1 µl of a 1:10 dilution of the product of the first PCR. Cycling conditions for the primers 16Sar‐16Scrur consisted of an initial denaturation at 94ºC for 2 min followed by 39 cycles of denaturation at 94ºC for 30 s, annealing at 58ºC for 35 s, extension at 72ºC for 40 s, and a final step of 7 min at 72ºC. Cycling conditions for semi‐nested PCR with primers 16Scruf‐16Scrur consisted of an initial denaturation at 94ºC for 2 min followed by 33 cycles of denaturation at 94ºC for 30 s, annealing at 56ºC for 35 s, extension at 72ºC for 40 s, and a final step of 7 min at 72ºC. PCR amplifications were carried out in a TGradient thermocycler (Biometra), and visualized on a 1.5% agarose gel.

Semi‐nested PCR products with the expected size (around 200 bp) were cloned using TOPO TA Cloning kit (Invitrogen) following the manufacturer's protocol. Five clones per copepod were sequenced using 200 ng of plasmid DNA and the universal primer T7. Sequences recovered from clone libraries were considered to be part of the same operational taxonomic unit (OTU) if there was <1% sequence divergence, allowing for intra‐specific variation and Taq polymerase errors. OTUs were compared to sequences found in GenBank using the BLAST algorithm to identify metazoan prey.

A phylogenetic tree was constructed to assign unknown sequences to the highest taxonomic level and to verify the BLAST identifications (Figure 1b). The tree contained all OTUs obtained from the copepods, together with the five closest matches of each OTU that were downloaded from GenBank. Sequences were aligned using MAFFT v5.7 with default settings (Katoh & Standley, 2013). A substitution model was selected under the Akaike information criterion as implemented in jModelTest2 (Darriba, Taboada, Doallo, & Posada, 2012). The HKY+I+G model was chosen to infer the evolutionary history using the maximum‐likelihood (ML) method. The analysis involved 62 nucleotide sequences with a total of 158 positions in the final dataset. Bootstrap probabilities with 1,000 replications were calculated to assess reliability on each node of the ML tree. Evolutionary analyses were conducted in MEGA6 (Tamura, Stecher, Peterson, Filipski, & Kumar, 2013).

### 2.3 Estimating the metazoan‐copepod link: how much carbon could be missing?

**Figure 2:**
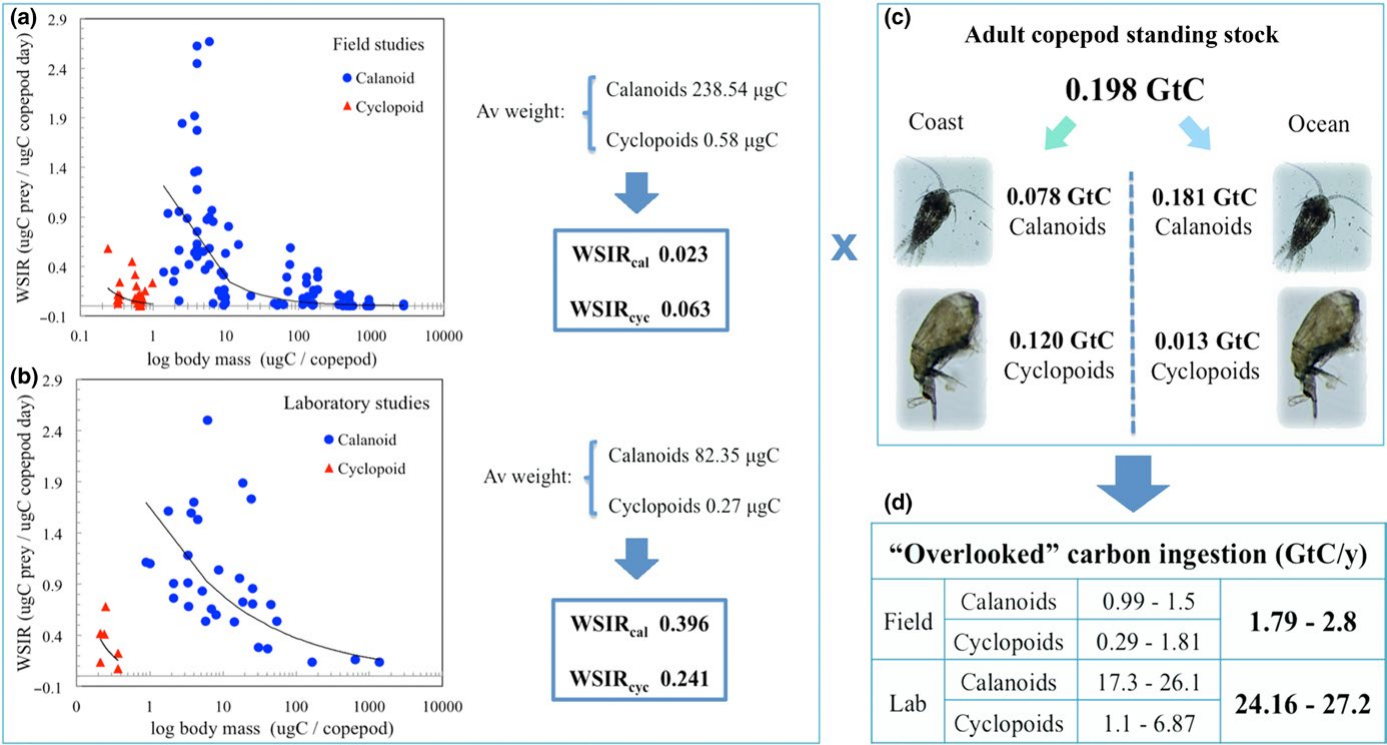
Global estimates of the metazoan‐copepod link. Weight specific ingestion rates (WSIR) of carnivorous and omnivorous adult calanoid (WSIR_cal_) and cyclopoid (WSIR_cyc_) copepods (Saiz & Calbet, 2007) were estimated using their average weight from field (a) and laboratory studies (b). Adult calanoid and cyclopoid copepod standing stocks in GtC were calculated using different calanoid‐cyclopoid percentages obtained for coastal (NRS‐IMOS database) and oceanic realms (COPEPOD database) (c). Estimated annual carbon ingestion through the metazoan‐copepod link (d), which results from multiplying the WSIR obtained in a and b by the adult copepod standing stock shown in c

We first obtained an estimate of the weight specific ingestion rates (WSIR) of adult calanoid and cyclopoid copepods that were fed carnivorous and omnivorous diets. The dataset used included predation on heterotrophic dinoflagellates, ciliates, and metazoans (Figure 2a,b) from field (*n* = 122) and laboratory (*n* = 37) studies (Saiz & Calbet, 2007), to obtain an average estimate of metazoan ingestion rates for small and large copepods. We assumed that 60% of the copepods at a given time are adults and older stages (Turner, 2004), and these are likely to feed on metazoans more than nauplii and younger stages. Using the mean weight of calanoids and cyclopoids as a proxy, we obtained an estimate of the ingestion rate for these two groups (Figure 2a,b). The greater variability in field studies, including experimental methods, food concentrations, and copepod assemblages present, results in greater variability in estimates for small copepods (5–10 µgC, Figure 2a). Since most laboratory studies are at near saturation conditions, the WSIR measured in the laboratory (Figure 2b) were between 17 and four times higher than those obtained in the field for calanoids and cyclopoids, respectively.

We then multiplied the WSIR by the adult copepod and older copepodite standing stock in the first 100 m of the water column globally (Figure 2c). This value was estimated using the following assumptions: zooplankton standing stock biomass in the upper 100 m = 0.31 gigatons of C (GtC) globally (Bogorov, Vinogradov, Voronina, Kanaeva, & Suetova, 1968; Moiseev, 1971). Biomass was corrected by increasing it by 1/3 since mesozooplankton biomass is underestimated with 200 μm nets (Gallienne & Robins, 2001). About 80% of the zooplankton biomass is copepods (Kiørboe, 1998) and ~60% of the copepods at a given time are adults and older copepodites (Turner, 2004). Given that calanoid and cyclopoid copepods displayed significantly different WSIR (Figure 2a,b), we then split the adult copepod biomass into calanoids and cyclopoids to better estimate their contribution to the total biomass. We used two datasets to obtain an approximation of the different percentages of calanoids and cyclopoids from coastal and oceanic environments (Figure 2c): (a) the coastal environment was represented by the National Reference Stations of the Integrated Marine Observing System (NRS‐IMOS: https://imos.org.au/home.html) where all the stations are situated over the continental shelf; and (b) the oceanic environment was represented by the Coastal and Oceanic Plankton Ecology, Production Observation Database (COPEPOD) a global coverage database of the National Marine Fish Service of USA (NMFS: https://www.st.nmfs.noaa.gov/copepod/).

We filtered both databases to keep only adult and copepodites collected above 100 m and with a fine mesh size (100–116 μm) to avoid underestimating their abundance (Gallienne & Robins, 2001). The coastal realm was represented by 60.5% calanoids, 28.5% cyclopoids, and 11% poecilostomatoids (NRS‐IMOS). On the other hand, 294,093 copepod records represented the oceanic realm from all around the world (COPEPOD), resulting in an average percentage of 91.3% calanoids, 5.9% cyclopoids, 2.4% harpacticoids, and 0.4% poecilostomatoids. The small poecilostomatoid genera *Corycaeus* and *Oncaea* are well known to be carnivorous (Turner, 2004) and have been traditionally considered cyclopoids and have recently been placed once again in this order, we included them in our calculations as cyclopoids. Finally, the product of WSIR by the adult copepod biomass in the upper 100 m was multiplied by 365 days to estimate the amount of carbon ingested through the overlooked metazoan‐copepod link annually (Figure 2d).

### 2.4 Impact of the metazoan‐copepod link in pelagic fluxes of C and N

**Figure 3:**
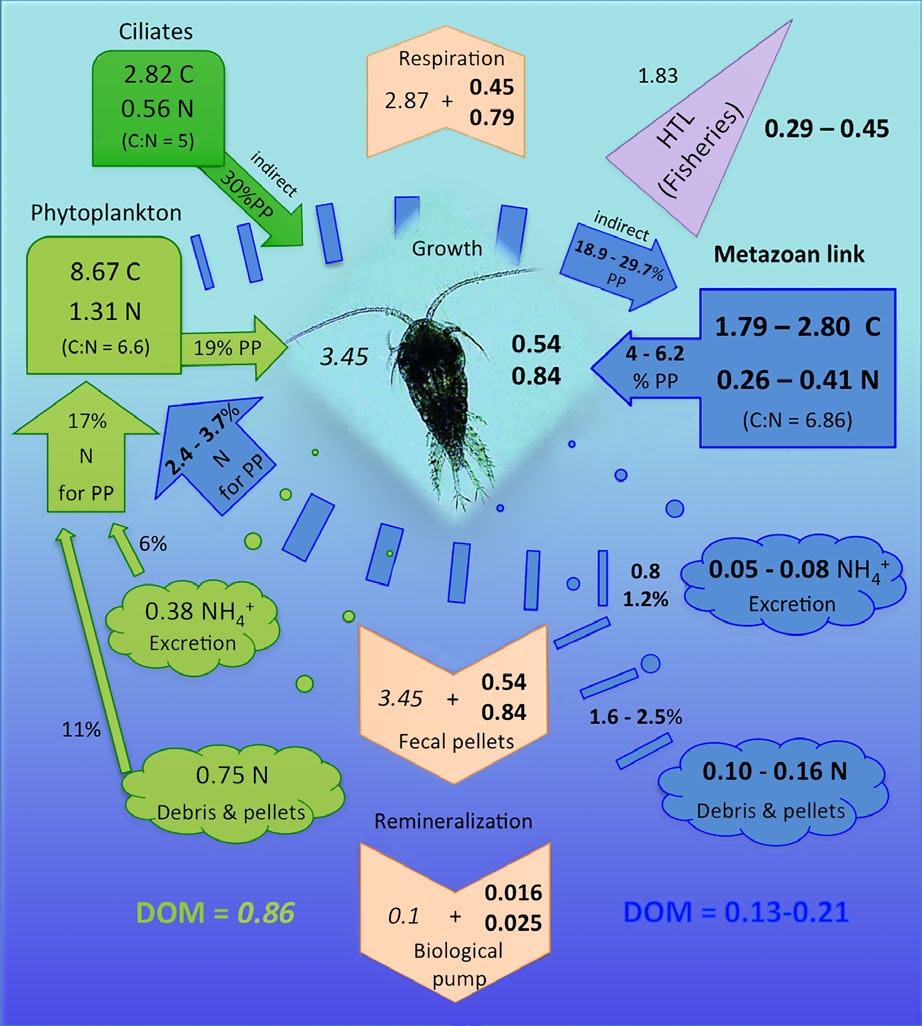
Potential global impact of the metazoan‐copepod link in pelagic fluxes of C and N expressed in gigatons per year in the top 100 m of the ocean. Field‐estimated contributions of the metazoan‐copepod link are shown in blue and the consequent increase in copepod respiration, growth, indirect primary production, ingestion, fecal pellet production, biological pump, and export to higher trophic levels (HTL) is represented in bold. Copepod predation on phytoplankton and ciliates (Calbet & Saiz, 2005) were calculated under the standing stock assumptions of this study (see Section 2 for details). Copepod growth, remineralization, dissolved organic matter (DOM), and transfer to HTL estimated from the COBALT model (Stock et al., 2014) are shown in italics

To estimate the consequences that this overlooked input of carbon could have on the trophic web in the top 100 m (Figure 3), fluxes of C and N were calculated using the following data: standing stock of phytoplankton = 45 GtC (Falkowski, Barber, & Smetacek, 1998). Values of C and N coming from phytoplankton and ciliate grazing (Calbet & Saiz, 2005), but corrected considering that the zooplankton biomass was underestimated by 1/3 (Gallienne & Robins, 2001) and 80% of that biomass is composed of copepods (Kiørboe, 1998). Key rates for copepods (Ikeda & Motoda, 1978): assimilation efficiency = 0.7, growth efficiency = 0.3, ingestion rate = 2.5 respiration, and growth rate = 0.75 respiration. Excreted NH_4_^+^ and N released in debris and pellets ~20% and 40% of ingested *N* (Kiørboe, Mohlenberg, & Hamburger, 1985). Global mean C:N of zooplankton = 6.86 (Martiny et al., 2013), C:N of phytoplankton = 6.6 (Ho, 2003), and C:N of ciliates = 5.0 (Stoecker & Capuzzo, 1990). The contribution of copepods to higher trophic levels (fisheries, 8%), remineralization (8%), and dissolved organic matter (DOM, 3%–5% of copepod biomass) were obtained from the COBALT model (Stock et al., 2014). Finally, carbon sinking was 3% of egestion (Turner, 2015).

## 3 RESULTS

### 3.1 Metazoan predation in copepods

Between one and four OTUs were detected within the digestive tracts of the copepod species investigated (Table 1). Most of the OTUs (10 of 16) showed homologies >96% with sequences available on GenBank and were identified to species level. The taxonomic identity of the remaining OTUs (78%–93% homology) was assigned on the basis of their topographical position on the consensus tree using bootstrap methods (Figure 1b). In total, 16 OTUs belonging to three different phyla were detected: Crustacea (*n* = 13), Mollusca (*n* = 2) and Chordata (*n* = 1). A total of seven and 12 OTUs were detected in the copepods collected under downwelling (*n* = 7) and upwelling conditions (*n* = 10), respectively (Table 1), but there was no significant difference in the mean number of prey detected for each species (1.86 ± 0.69 for upwelling and 2 ± 1.15 for downwelling, *t* test: *p* = 0.387).

Within the crustaceans, eight different families of decapods were identified: crabs (Pirimeliidae, Polybiidae and Goneplacidae), porcelain crabs (Porcellanidae), snapping shrimps (Alpheidae), ghost shrimps (Callianassidae) and mud shrimps (Upogebiidae, Laomediidae), as well as the euphausiid *Nyctiphanes couchii*. Unexpectedly, copepod predation was detected in the small copepod *Diaixis pygmaea*, where the DNA of the copepod *Metridia lucens* was found. The invasive mytilid bivalve *Limnoperna securis* was detected within the digestive tract of the copepod *Centropages typicus*, as well as another mytilid not present in the genetic database. Finally, a fish belonging to the subfamily Gobiinae was detected in the calanoid copepod *Isias clavipes*. The crab *Pirimela denticulata* was detected in the cyclopoid copepod *Oithona* sp., while an undefined snapping shrimp (Alpheidae) was detected in the poecilostomatoid *Corycaeus* sp.

### 3.2 Estimating the metazoan‐copepod link: how much carbon could be missing?

Based on copepod diets that include predation on heterotrophic dinoflagellates, ciliates and metazoans and mean copepod mass of calanoids and cyclopoids, we estimated that the WSIR was 0.023 and 0.063 for field studies (*n* = 122) and 0.396 and 0.241 µgC prey/µgC copepod per day for laboratory studies (*n* = 37), using the mean body mass of calanoids and cyclopoids, respectively (Figure 2a,b). The estimated standing stock of calanoid/cyclopoid copepods in the first 100 m was 0.12/0.078 GtC for the coastal area and 0.181/0.013 GtC for the ocean (Figure 2c) based on the percentages obtained from the two large public datasets.

Assuming the WSIR obtained as an estimate of the metazoan‐copepod link, then multiplying the WSIR by the copepod standing stock and converting to an annual estimate (Figure 2d) we calculate that: (a) 1.79–2.80 GtC/year would be ingested by calanoid and cyclopoid copepods in oceanic and coastal realms, respectively, using the WSIR estimated from field studies; and (b) this figure would increase up to 24.16–27.20 GtC/year if we use the WSIR estimated from laboratory studies.

### 3.3 Impact of the metazoan‐copepod link in the pelagic fluxes of C and N

Estimates from field studies (1.79–2.80 GtC/year, Figure 2d) were used to examine the hypothetical impact of the metazoan‐copepod link in terms of fluxes of C and N in the pelagic trophic food web (shown in blue in Figure 3). Within this preliminary global‐scale estimate, the metazoan‐copepod link would represent 4.0%–6.2% of the primary production. This pathway of carbon and nutrients could support an equivalent 15.6%–24.4% increment in copepod growth, pellet production, and carbon sinking to deeper layers through the biological pump, as well as more carbon exported to higher trophic levels. The amount of N excreted through ammonia or fecal pellets would increase the nutrients available to PP in the photic layer from 17 to 20.7. Another indirect consequence of this disregarded trophic link is the amount of PP that indirectly reaches copepods through the consumption of zooplankton (Figure 3). Thus, assuming that their zooplankton prey mostly feed on phytoplankton through a single trophic level, then 18.9%–29.7% of PP would be assimilated by the zooplankton and ultimately transferred to copepods.

## 4 DISCUSSION

Copepods are the most abundant metazoan animals on Earth and are a key link in pelagic ecosystems, preying upon unicellular organisms, and serving as prey for higher trophic levels (Gattuso et al., 2015; Pauly et al., 2002). Our preliminary results support that this current paradigm of “herbivorous” copepods could be underestimating their true role in the marine ecosystem by missing a key energy source. The molecular technique applied here showed that all the copepods analyzed from a coastal upwelling systems under two contrasting oceanographic conditions (upwelling vs. downwelling) had metazoan prey within their guts. Using a meta‐analysis of published copepod feeding rates, we estimated that such metazoan‐copepod link at global scale could be 1.79–27.20 GtC/year. This source of carbon—currently missing in global ecosystem models—could have cascading effects in the pelagic environment, increasing current estimates of biogeochemical fluxes (remineralization, respiration, and biological pump among others) as well as increased export to higher trophic levels.

Despite the relatively low number of clones sequenced and the rapid digestion of prey in copepods (Kleppel, 1993), we were able to detect 1–4 OTUs in the digestive tract of all copepods analyzed, suggesting that ingesting metazoans is common for small copepods and may be a significant component of their diet. Overall, up to 16 different prey/OTUs were detected in the dominant copepod community of the coastal upwelling region of NW Iberian Peninsula. With the advent of next generation sequencing (NGS) technologies, this molecular methodology can be successfully adapted with other universal primers targeting phytoplankton, ciliates, and flagellates to gain a deeper insight into the copepod trophic relationships (e.g. Cleary et al., 2015). Recently, a slight modification of the primers used in this work was applied in a NGS study to detect prey in planktonic cephalopod paralarvae revealing up to 122 molecular OTUs (Olmos‐Pérez, Roura, Pierce, Boyer, & González, 2017).

Unfortunately, molecular methods cannot distinguish developmental stages of the prey ingested (eggs, nauplii, larvae, or adults) and this can have repercussions in terms of carbon ingested depending on the size of the prey. Considering that the mean size of the copepods analyzed in this study was close to 1.5 mm total length, it is likely that the majority of metazoan prey ingested would be early stages (e.g. nauplii, zoeae, veliger). Nonetheless, the physical limitations involved in swallowing prey larger than the mouth would involve sloppy feeding, which is the breakage and partial ingestion of the prey, releasing particulate, and dissolved carbon to the water column (Møller, 2005). Despite the partial ingestion of the metazoan prey, the amount of carbon incorporated into copepod biomass could range from one to three orders of magnitude higher than that of preying upon ciliates/flagellates and phytoplankton (Saiz, Griffell, Calbet, & Isari, 2014).

Despite the high sensitivity of molecular techniques that allow detection of small quantities of DNA, little is known of the origin of the DNA (King, Read, Traugott, & Symondson, 2008). We cannot be certain whether copepods directly prey upon metazoans or upon fecal pellets of other metazoans (secondary predation through scavenging). Field studies provide some support for predatory behaviour in some small copepods as crustacean mandibles, fish eggs, and fish larvae have been observed within their guts and fecal pellets (reviewed in Kleppel, 1993, Turner, 2004). Secondary predation is a potential source of error when tracking trophic links (Sheppard & Harwood, 2005) and may be significant in copepods owing to their feeding strategy. However, since cyclopoid copepods are known to feed on fecal pellets where DNA is highly degraded (Turner, 2004), and detecting secondary predation heavily decreases with increasing time since ingestion (Sheppard & Harwood, 2005), the likelihood of detecting prey DNA from a scat within a copepod would be expected to be low compared with that of a recently consumed prey item. It is thus more likely that the DNA detected within the digestive tract of the copepods originates from fresh metazoans (either predated or grazed) rather than from scavenging (fecal pellets, marine snow or molts).

Although it has been assumed that in productive environments the linear diatom‐copepod‐fish food chain predominates after an upwelling event (Kleppel, 1993), it is now evident that most primary production is consumed by microzooplankton and small metazoans (Mitra, Flynn, et al., 2014), with diatoms contributing only up to 8% of the copepod diet globally (Mitra, Castellani, et al., 2014). We expected that metazoan predation by copepods would be higher under downwelling conditions, since less primary production was available and they would have to switch to carnivorous feeding (Kleppel, 1993). In a recent study, the relative frequency of metazoan prey—measured as the relative abundance of mesozooplankton or gelatinous plankton sequences—detected in the small *Pseudocalanus* species was higher under low chlorophyll concentrations (<2 μg/L, equivalent to the downwelling conditions of our study), than under medium (4–6 μg/L) or high chlorophyll concentrations (>20 μg/L, Cleary et al., 2015). However, in our preliminary study, there was no significant difference in the mean number of prey detected per copepod species under downwelling or upwelling conditions. This result might be explained due to the low number of OTUs analyzed per copepod, as well as the low number of copepods analyzed.

Our study suggests that trophic pathways in upwelling ecosystems involve complex, highly interactive webs (Figure 1c), instead of the conventional linear trophic chain. Moreover, the metazoan‐copepod link detected is bidirectional, not only affecting the mortality and export to higher trophic levels but also promoting PP by releasing nutrients through sloppy feeding and reducing grazing pressure (Mitra, Castellani, et al., 2014). The metazoan‐copepod link (Figure 2d) represents a 15.6%–24.4% increase in the ingestion of carbon by small copepods compared to the combined ingestion of phytoplankton and ciliates (8.67 and 2.82 GtC/year, respectively, Calbet & Saiz, 2005).

Feeding rates used in the calculations in Figure 2 included values for copepods consuming ciliates and flagellates—where copepods are most efficient—but also large metazoan prey that may represent just one capture per day. Despite marked differences in the ingestion rate of these organisms, we assumed a mean carnivorous ingestion rate for all copepods (small and large), as estimates would be unrealistically underestimated if we only considered ingestion rates obtained for large copepods feeding upon large metazoans. Optimal prey/predator ratios reported for suspension‐feeding calanoid copepods (2%–6%) and cyclopoids (4%–10%) (Saiz et al., 2014) suggest that small copepods (<2 mm) might optimally capture prey ranging from <0.04 mm up to 0.2 mm, and even upon prey larger than themselves such as fish larvae (Calbet, Carlotti, & Gaudy, 2007). Therefore, we have been conservative by including ingestion rates obtained for smaller copepods feeding upon unicellular organisms and small metazoans. This enabled us to obtain a mean feeding rate that includes the “carnivorous” repertoire of copepods ranging from small nauplii to large fish larvae.

Taking into account the difficulties of obtaining a mean feeding rate for copepods that display an broad variety of feeding behaviors (ambush predators vs. suspension feeders) and the capability to switch between them (Kiørboe, 1998) across a wide range of sizes (from <0.5 mm to >1 cm), the results obtained in this study suggest remarkable consistency between our field estimates (1.79–2.80 GtC/year, Figure 2d) and those obtained with the ecosystem model COBALT (Stock et al., 2014), where 2 GtC/year of small copepods (<2 mm) are ingested by the larger mesozooplankton fraction (>2 mm). Also notable is that the carbon ingestion estimated here for the top 100 m based on laboratory studies (24.16–27.20 GtC/year, Figure 2d) is similar in magnitude to the 26 GtC/year estimate based on mesozooplankton community respiration (Hernández‐León & Ikeda, 2005). This figure is surprisingly close to that estimated by Buitenhuis et al. (2006), who suggested that up to 25.8 GtC/year of direct and indirect grazing on particulate organic matter (POC) and bacteria would have to make up the largest part of mesozooplankton predation to reconcile the grazing flux obtained in their optimized model of 42.8 GtC/year.

The metazoan‐copepod pathway appears to represent a significant input of carbon (Figure 2d) that could account for the discrepancies usually found between quantified unicellular ingestion and metabolic demands of copepods, supporting production rates and survival when there is limited available food in terms of phytoplankton (Calbet, 2001; Hirst & Bunker, 2003; Mayor, Sommer, Cook, & Viant, 2015). It will also help to clarify discrepancies found in global models between the observed and modeled copepod biomass. If the copepod‐metazoan link would be included in these models, 15.6%–24.4% more biomass could be available for copepod production (Figure 3) and there would be no need to artificially increase the copepod grazing rates on bacteria (Buitenhuis et al., 2006) and phytoplankton (Hernández‐León & Ikeda, 2005) or consider an “artificial” (i.e. largely below the optimal prey/predator ratio) carbon ingestion coming from microzooplankton (Stock et al., 2014).

The complex interaction between bottom‐up and top‐down effects currently hampers our capacity to predict how the inclusion of the metazoan‐copepod link will affect climate change predictions. Nonetheless, our preliminary calculations show that a more realistic interpretation of copepod trophic interactions could uncover a carbon pathway that has either been overlooked or thought to be minor (Buitenhuis et al., 2006; Kiørboe, 1998; Ohman & Hirche, 2001), with cascading effects across the entire pelagic and benthic ecosystems increasing current estimates of biogeochemical fluxes (remineralization, respiration, and biological pump) and export to higher trophic levels by 15.6%–24.4% (Figure 3). The metazoan‐copepod link is roughly quantified here and requires further investigation. If further studies confirm its magnitude as suggested here, the metazoan‐copepod link needs to be accounted for in global ecosystem to represent accurately the zooplankton component_._ This could ultimately enhance our capacity to predict the fate of increased anthropogenic emissions in the oceans likely impacting current predictions of climate change or fisheries yields.

## AUTHORS’ CONTRIBUTIONS

AR conceived and designed the experiment, performed the experiments, wrote the first draft of the manuscript; AR and AFG collected samples; AR and AJR analyzed the data; AR, JMS, AFG, and AJR contributed reagents/materials/analysis tools; and all authors contributed substantially to revisions.

## DATA ACCESSIBILITY

Prey detected in copepods is available on GenBank under accession numbers FR851241–FR851253 and LN614707–LN614709. Copepod data were obtained from the public databases NRS‐IMOS and COPEPOD.
